# Macromolecular Conjugate and Biological Carrier Approaches for the Targeted Delivery of Antibiotics

**DOI:** 10.3390/antibiotics6030014

**Published:** 2017-07-04

**Authors:** Nhan Dai Thien Tram, Pui Lai Rachel Ee

**Affiliations:** Department of Pharmacy, National University of Singapore, 18 Science Drive 4, Singapore 117543, Singapore; nhan.tram@u.nus.edu

**Keywords:** targeted delivery, antibiotic resistance, macromolecular conjugate, prodrug, erythrocyte carrier, bacteriophage carrier

## Abstract

For the past few decades, the rapid rise of antibiotic multidrug-resistance has presented a palpable threat to human health worldwide. Meanwhile, the number of novel antibiotics released to the market has been steadily declining. Therefore, it is imperative that we utilize innovative approaches for the development of antimicrobial therapies. This article will explore alternative strategies, namely drug conjugates and biological carriers for the targeted delivery of antibiotics, which are often eclipsed by their nanomedicine-based counterparts. A variety of macromolecules have been investigated as conjugate carriers, but only those most widely studied in the field of infectious diseases (e.g., proteins, peptides, antibodies) will be discussed in detail. For the latter group, blood cells, especially erythrocytes, have been successfully tested as homing carriers of antimicrobial agents. Bacteriophages have also been studied as a candidate for similar functions. Once these alternative strategies receive the amount of research interest and resources that would more accurately reflect their latent applicability, they will inevitably prove valuable in the perennial fight against antibiotic resistance.

## 1. Antibiotic Resistance and the Pressing Need for Alternative Therapeutic Strategies

The groundbreaking discovery by Sir Alexander Fleming marked the beginning of the epoch of antibiotics, during which uncountable human lives were saved [1,2]. Unfortunately, inappropriate prescribing and misuse of antibiotics in not just developing but also developed countries have been fueling the steady rise of antibiotic resistance worldwide [3,4,5]. The problem was further aggravated by uncurbed use of antibiotics in the environment [6]. Reports by multiple major healthcare agencies have highlighted this dismal trend, and called for measures to address the situation [7]. Many bacterial strains have developed resistance to multiple antibiotics. For instance, methicillin-resistant *Staphylococcus aureus* (MRSA) is resistant to, apart from methicillin, a host of common antibiotic classes, namely aminoglycosides, macrolides, tetracycline, chloramphenicol, and lincosamides [8,9]. To worsen the matter, particular strains have developed resistance to so many antibiotic agents that new technical terms had to be coined to describe them more accurately (“extensively drug resistant” and “pandrug-resistant” [10]). In May 2016, a strain of *Escherichia coli* in the United States was reported to be resistant to colistin, the last resort antibiotic [11]. Right from the onset of the antibiotic era, Sir Alexander Fleming himself warned the community about the occurrence of penicillin resistance and probable implications [12]. If only the sheer gravity of his cautionary advice was heeded earlier and necessary actions were taken accordingly, the current prospect would not have looked so grim.

Although the rapid emergence of multidrug resistance presents a grave public health concern, the development of novel antibiotics has steadily declined [13]. Very few antibiotics reached the market in the last decade. A number of economic and regulatory hurdles have discouraged pharmaceutical companies from channeling resources into antimicrobial research [1,3]. In fact, many large companies have abandoned the antibiotic sector altogether [6]. 

Confronted with a dwindling global pipeline for new antibiotics, it is high time we adopt alternative approaches to combat antibiotic resistance. Targeted delivery is one of the predominant tactics adopted by researchers. This direction aims to selectively deliver antibiotics to the targeted bacteria at the site of infection. This allows most of the drug to reach the bacteria where they can best exert their therapeutic effect while minimizing collateral damage exerted on the rest of the patient body. Some species of bacteria develop resistance by hijacking and inhabiting human host cells. Antimicrobial agents effectively bactericidal against those strains in vitro could become ineffectual in vivo due to poor accumulation inside the host cells [14]. Therefore, perpetual exposure to a sub-therapeutic concentration of the antibiotic naturally fosters the development of resistance by the pathogen [15]. Under such circumstances, selective release of the bactericidal agent inside these cellular niches will help ensure the desired amount at the desired site, thereby addressing the issue of antibiotic resistance.

## 2. Nanoparticle-Based Strategies for Targeted Delivery: A Brief Update

In principle, enhanced permeability and retention (EPR), a phenomenon extensively studied and rigorously exploited by nanomedicine for passive targeting in the advancement of cancer chemotherapeutics [16], could also be found at infected sites [17]. Heightened angiogenic activity and increased vascular permeability are the hallmark of infection-induced inflammation [18]. As such, efforts have been made to adopt passive targeting strategies into the field of antimicrobials. A variety of nanosized materials have been investigated as carriers for different classes of antibiotic agents (e.g., liposome [19,20,21,22,23,24], polymeric nanoparticles [25,26,27,28,29], solid lipid nanoparticles [30,31,32,33,34,35], dendrimer [36,37,38,39,40]). Nanocarriers have also been demonstrated to be capable of simultaneously delivering multiple antibiotics from different classes whose mechanisms of action complement one another, thereby producing a synergistic antimicrobial effect when present together at the site of infection [41].

Aside from enabling site-specific release of therapeutics, nanoparticles have been investigated for their intrinsic antimicrobial properties, regardless of the efficacy of their antibiotic cargo [42]. Notable advantages include relatively cost-efficient synthetic process, satisfactory stability upon storage, and the capability to tolerate substantial changes in physical conditions such as high temperatures employed during sterilization [43]. In addition, the materials investigated thus far have not been reported to cause significant acute adverse effects, unlike traditional antibiotics [44]. Whether any undesirable effects could surface in the long term, however, remains to be investigated. The majority of nanoparticles examined are inorganic (e.g., silver [45], titanium oxide [46], zinc oxide [47], copper oxide [48], iron oxide [49], gold [50]). That being said, organic nanomaterials have also been considered [42,44]. Not all of them exhibited sufficiently potent antibacterial capacity, and required modifications to become more applicable [51]. Interestingly, a number of these antimicrobial nanoparticles have displayed synergism with traditional antibiotics. Synergism was evident when silver nanoparticle was tested in combination with penicillin G, ampicillin, erythromycin, chloramphenicol, or vancomycin against both Gram-positive and Gram-negative bacteria [52,53].

A number of nanoparticle carrier constructs of antibiotics have reached clinical trials. For instance, Arikace^TM^, a lung-targeted liposomal formulation of amikacin for treating *Pseudomonas aeruginosa* chronic infection in cystic fibrosis patients, have passed phase I and phase II in clinical trials [54].

**Figure 1:**
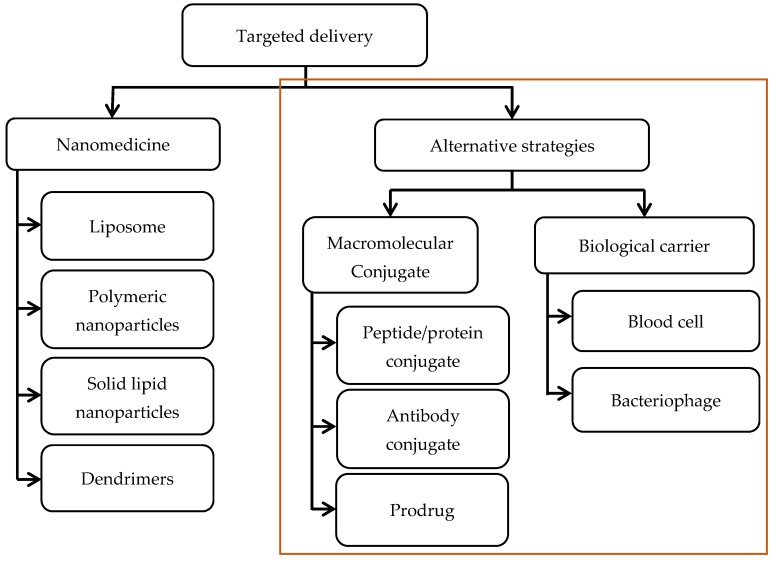
Overview of the different strategies available for developing targeted delivery systems of antibiotics. The focus of the article is on the alternative strategies which do not employ nanoparticles.

There have already been numerous well-documented reviews on the use of nanosized carriers for targeted delivery of antibiotics that readers may refer to [44,55,56,57]. The primary scope of this review will instead be other innovative strategies for targeted delivery that make use of conjugates between therapeutics and biochemical compounds, or chemical moieties, or even cells, but not nanoparticles (Figure 1).

## 3. Macromolecular Conjugate Strategies

Conjugating antibiotics to macromolecules is not a recent innovation in pharmaceutical research [58]. Initially, the strategy was employed to optimize physicochemical properties of drug compounds in order to improve their performance in vivo. Back in the 1990s, there was already an attempt to link polyethylene glycol (PEG) to amphotericin B and polymyxin [59]. Another study demonstrated that the circulating half-life of bacterial endopeptidase could be prolonged upon being conjugated with poly(alkylene oxides) [60]. Eventually, macromolecular conjugates were also recognized for their potential as delivery vehicles of antibacterial drugs.

**Figure 2:**
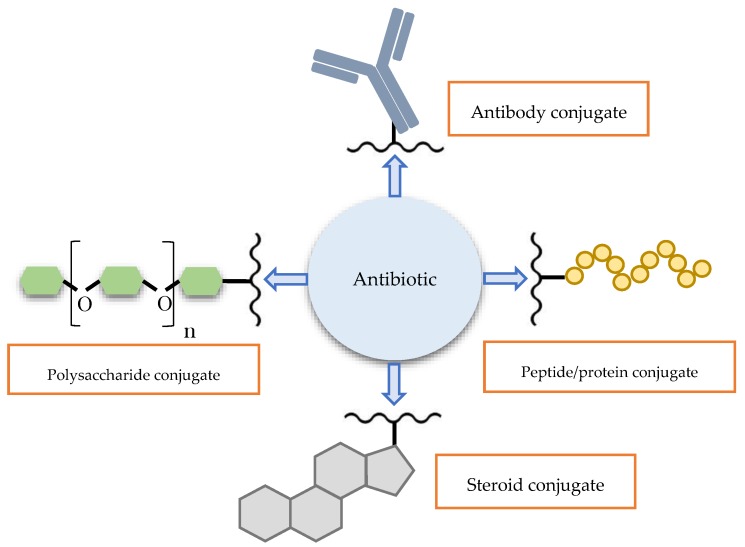
Schematic illustration of major types of macromolecules that could potentially form conjugates with antibiotics for targeted delivery.

One of the pioneering inventions in this field claimed a structure that covalently bonded amoxicillin to asialoganglioside, a receptor that selectively binds microorganisms [61]. The receptor acted as a homing device that helped direct the penicillin drug towards body parts with an abundance of bacteria. As a result, the efficacy of amoxicillin in treating *Streptococcus pneumoniae* and *Helicobacter pylori* infections was enhanced. From this starting line, the field grew rapidly to take into consideration other candidates for conjugation, ranging from polysaccharide, steroid, antibody, to peptide and protein (Figure 2). This article will explore the more widely studied groups of antibiotic conjugates.

### 3.1. Conjugate with Peptide/Protein

The general structure of a protein conjugate, or any macromolecular conjugate for that matter, consists of an antibacterial agent, a linker, which may be dispensable, and the macromolecule with the intended targeting function.

In 1982, *N*-formyl methionyl tripeptide and tetrapeptide, sequences reported to display a remarkable level of chemotactic activity for leukocytes and monocytes, were conjugated to silver sulfadiazine to amplify its effectiveness in treating bacterial infection at burn wounds [62]. Silver sulfadiazine exerts antibacterial activity, but impedes leukocyte chemotaxis to the infected region [63]. Conjugation with *N*-formyl methionyl peptides helped attenuate this undesirable effect of the agent. Although this is not by any means a typical example of a targeted delivery system, it introduced the concept of affinity between the conjugated segment and the target cells, and laid the groundwork for later studies in the field.

Ovotransferrin was conjugated to sulfa antibiotics to direct the drugs towards bacteria and human epithelial cells by virtue of its affinity to the transferrin receptor found on the membrane surface of target cells [64,65]. Ovotransferrin belongs to the transferrin family, a group of glycoproteins that bind iron [66]. Its structure comprises hydrophobic regions consisting of aromatic residues that readily form non-covalent interactions with aromatic rings, which are prevalent among common antibiotics [67]. Evidence from UV absorption showed that the antibiotics were enclosed inside the conjugate. Moreover, disulfide moieties in the conjugate are particularly vulnerable to attacks by reducing agents. This attribute is beneficial since it allows the bonds to be severed and drug cargo to be liberated from the drug–protein conjugate once it comes in contact with either the bacterial membrane or the endosome, both of which are redox-active. Consequently, the construct is not only capable of targeted delivery, but also the ability to selectively release the antibiotic once inside bacterial cells.

On its own, ovotransferrin displayed bactericidal effect attributable to the antimicrobial peptide domain OTAP-92 on its surface [68]. OTAP-92 was reported to be effective against both Gram-positive and Gram-negative bacteria by damaging their plasma membrane [69]. In fact, ovotransferrin was evidently more potent against *Propionibacterium acnes*, and *Corynebacterium minutissimum* than tested antibiotics [67]. Multidrug-resistant *Salmonella enteritidis*, while largely resistant to either ovotransferrin or sulfabenzamide, was shown to be susceptible to the conjugate between the two.

Hai et al. investigated the efficacy of the conjugate between apotransferrin and amoxicillin against *Chlamydia trachomatis* [70]. This microbe is an obligate intracellular pathogen that flourishes inside host vacuoles while being safeguarded from therapeutics by multiple layers of host cell membranes [71]. Its unique habitat has proven to be a real challenge to the development of successful therapies [72]. Using immunofluorescence microscopy, it was illustrated that apotransferrin, the targeting ligand of interest, readily entered cells infected by *Chlamydia trachomatis* and co-localized with the chlamydial inclusions [70]. Apotransferrin-amoxicillin conjugate exhibited substantially stronger anti-chlamydial effect than unconjugated drug, thereby suggesting that the transferrin species helped direct amoxicillin to the chlamydial inclusion membranes inside the host cells.

**Figure 3:**
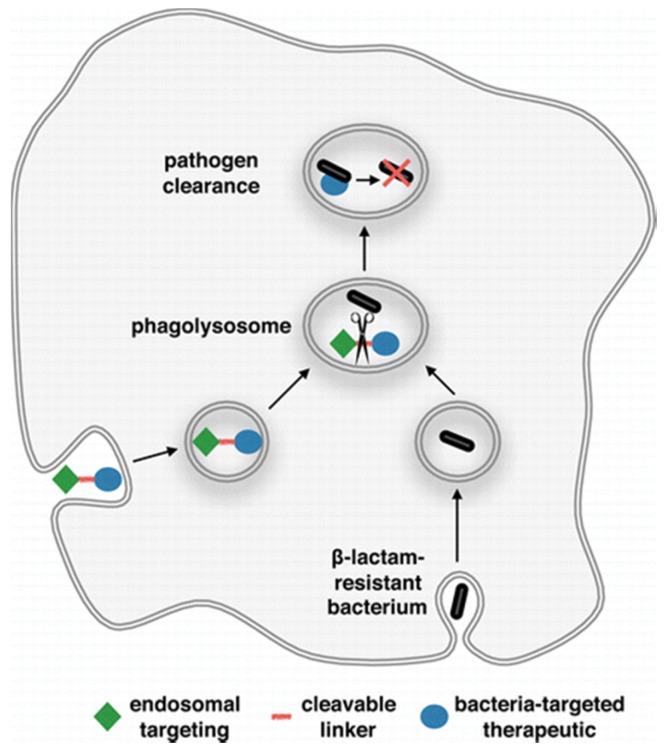
Mechanism of selective release of methotrexate prodrug inside the macrophages, which exploits the β-lactamase produced by mycobacteria (reprinted with permission from Pereira et al. [73], Copyright © 2015, American Chemical Society).

A cationic and hydrophobic peptide was conjugated to methotrexate to improve its ability to pass through the mycolic acid layer in the cell wall of *Mycobacterium smegmatis* and *Mycobacterium tuberculosis* (Figure 3) [73]. Cephalosporin was included as the cleavable part of the linker in order to exploit the β-lactamases expressed by mycobacteria [74]. The targeting peptide provided an additional benefit of directing the bioconjugate towards mitochondria once inside human cells [75]. This prevented methotrexate from inhibiting human dihydrofolate reductase, which occurs in the cytosol and the nucleus [76]. Therefore, the peptide played a vital role in reducing collateral toxicity of the drug on macrophages.

Surolia et al. explored the utility of transferrin as a targeting device for the treatment of *Plasmodium falciparum* [77]. In place of a traditional antibiotic agent, immunotoxins constituted the antibacterial part of the conjugate. They are commonly formed from ricin and *Pseudomonas* exotoxin. Diphtheria toxin is another extensively studied toxin. Such hybrid constructs are endowed with both the antimicrobial activity of the toxin as well as the targeting affinity of the protein [78]. Trophozoites and schizonts of *Plasmodium falciparum* produce antigens that are expressed on the surface of infected erythrocytes. One of them is a transferrin receptor, and this is the primary reason why transferrin was selected as the targeting moiety. An additional advantage of this targeting strategy lies in the structural difference between transferrin receptors found on the surface of infected red blood cells and those on host cells [79]. Conjugating transferrin to ricin A elevated its efficacy against chloroquine-resistant strain of *Plasmodium falciparum* [77]. Exposing the parasites to the conjugate halted their growth at the schizont stage, thereby preventing them from infecting newly introduced red blood cells.

Another non-traditional antibiotic investigated in a protein conjugate was phosphorodiamidate morpholino oligomer, a DNA analogue that kills bacteria by complementarily bind to specific gene sequences in the microbe genetic materials and disrupt their functions [80]. The oligomer has been demonstrated to be efficacious as an antibacterial against an assortment of both Gram-positive and Gram-negative bacteria (e.g., *Escherichia coli* [81], *Salmonella* [82], *Neisseria gonorrhoeae* [83], *Klebsiella pneumoniae*, and *Acinetobacter baumannii*). One major setback is its poor entry into bacterial cells [84]. To circumvent this, Mellbye et al. conjugated the oligomer to sequences of amphiphilic peptide alternating between cationic and non-polar segments [83]. Although the cationic peptide did not serve as a homing device, it helped improve antibacterial efficacy of the oligomer both in vitro and in vivo. A similar construct was adopted to enhance the efficacy of phosphorodiamidate morpholino oligomer in treating infections caused by *Burkholderia cepacia* complex in patients with chronic granulomatous disease and cystic fibrosis, which in general poses a challenge due to the emergence of antibiotic resistance [85].

### 3.2. Conjugate with Antibody

This strategy drew inspiration from the antibody–drug conjugate concept widely researched for the targeted delivery of cytotoxic chemotherapeutics to tumor sites [86]. Once again, the general structure of the conjugate comprises the antibiotic cargo, a linker that plays a more major role than in a protein conjugate, and the monoclonal antibody chosen to selectively guide the construct to the bacteria [87]. The conjugation step could be realized via several different methods such as alkylation of reduced disulfides [88], alkylation of genetically modified cysteine residues, and acylation of lysine residues [89]. The technique choice depends on the structure and the peptide sequence of the antibody of interest, and consequently what amino acid residues are found in a location and orientation favorable for forming bond with the linker. Since each monoclonal antibody most likely contains multiple residues ready to be linked, a heterogenous mixture of conjugate constructs with a variation of antibiotic density and localization is normally obtained. It has been recognized that the amount of antibacterial compound per antibody vehicle affects not only the pharmacokinetic behavior, but also the bioactivity of the conjugate [90].

The single most vital feature of the ideal linker is stability in circulation. Afterall, a well-designed bio-vehicle needs to be able to avoid offloading its cargo before reaching the target destination. At the same time, the ideal linker needs to be readily cleavable upon exposure to biochemical milieu inside endosomes or lysosomes in order to set the antibiotic free. Both of these properties work in tandem to ensure selective drug release. Typical designs of a cleavable linker exploit the discrepancy in pH level between endosomal or lysosomal interiors (pH 4.5–6.5) and the circulation (pH 7.3–7.5). Chemical moieties such as hydrozones and disulfides are selectively hydrolyzed at a slightly acidic pH [91]. The linker does not have to be exclusively cleaved under intracellular environment. It could also be designed to be severed by enzymes specific to bacterial biofilms [87]. 

Another parameter to be considered is the hydrophobicity of the linker. Since the majority of traditional antibacterial agents have a considerable level of hydrophobicity, attaching the cargo to a hydrophobic linker molecule could potentially engender the issue of self-aggregation by the conjugates [92]. Apart from the impairment of drug release mechanism, the aggregates could trigger host immunologic reactions [93].

Lehar et al. conjugated rifalogue, a rifamycin derivative, to anti-*Staphylococcus aureus* antibody for the treatment of MRSA [94]. The antibody specific to the wall-teichoic acids found in *Staphylococcus aureus* cell wall was attached to rifalogue using a linker cleavable by cathepsins. More recent inventions by Genentech Inc. (South San Francisco, CA, USA) also utilized antibodies against wall-teichoic acid as the targeting moiety [95,96]. Even though *Staphylococcus aureus* is not an obligate intracellular pathogen, it could form depots inside host cells, which, in many cases, contribute to the failure of antibiotics in completely eradicating bacteria from the patient’s body. Those secluded groups of bacteria will eventually re-enter the circulation, get distributed by the bloodstream, and cause a relapse of the infection. This ability of pathogens to survive antimicrobial drugs by seeking refuge inside host cells constitutes a therapeutic challenge [97], alongside the multidrug-resistance status of MRSA. 

By virtue of its size, the antibiotic–antibody conjugate did not indiscriminately diffuse across plasma membranes of mammalian cells [94]. Instead, it attached itself to the bacteria in the bloodstream, and was carried along with the pathogen when they entered the host cells. Once inside, the linker was cleaved to liberate the therapeutic cargo. Both of these behaviors ensured the targeted delivery functionality of the construct. It was demonstrated that conjugation substantially improved killing of MRSA inside all types of human cells tested, namely macrophages, endothelial and epithelial cell lines.

Granzyme B is a serine protease secreted by natural killer cells [98]. It has been shown in vitro to be capable of causing the destruction of red blood cells infected by *Plasmodium falciparum* [99]. Drawing inspiration from this finding, Kapelski et al. examined the efficacy of a conjugate between granzyme B and merozoite surface protein 4 (MSP4)-specific single-chain Fv protein, which helped direct the antimicrobial protease towards infected erythrocytes [100]. This target protein is expressed on the surface of malarial merozoites, and displays structural features optimal for targeting by antibodies [101]. It was evident that conjugation to the antibody enhanced the efficacy of granzyme B. A significantly lower IC50 value obtained when tested against *Plasmodium falciparum* multidrug-resistant strain K1 [98].

### 3.3. Prodrug

Although a prodrug is structurally distinct from a bio-conjugate, it can be argued that the two strategies share a fundamental similarity in the way both involves attaching a biochemical extension via a bond eventually cleaved at the desired target site. Therefore, the prodrug approach will be discussed in this article as a subgroup under the scope of conjugate strategies. At the earlier stage, the approach primarily involved affixing to the active drug molecule either a hydrophilic moiety to enhance water solubility for better dissolution in the gastrointestinal tract [102], or a hydrophobic moiety to facilitate passive diffusion across biological membranes [103]. Those original prodrug schemes were devoid of any target-specificity element, and thus were not suitable for targeted delivery.

In a more pertinent design, mannose was utilized as the targeting-moiety in a pyrimethamine prodrug for treating leishmaniasis [104]. Mannose receptors are expressed by human macrophages to identify microbes with proteoglycans in their membrane for phagocytosis [105]. Hence, mannose conjugation enhanced co-localization of the construct with the pathogen inside the macrophages. Dextran was incorporated as a polymer carrier because it was unlikely to trigger immune responses from the host [106]. The prodrug was devised to release the antiprotozoal agent after being endocytosed into the macrophages [107]. Both carboxymethyldextran-thiomannopyranoside-pyrimethamine and succinyldextran-thiomanno-pyranoside-pyrimethamine constructs yielded very good therapeutic efficacy against *Leishmania amazonensis* amastigotes nestled inside macrophages [104]. At the same time, the prodrugs did not afflict any noticeable damage to the host cells.

A different study also investigated the use of mannose and dextran to improved antimicrobial activity of norfloxacin against intracellular *Mycobacterium bovis* [108]. The selective drug release mechanism employed α carbon bond that would be rapidly cleaved by cathepsin B in the lysosome. Free norfloxacin could only exert its antimicrobial activity against the pathogen in vitro, but not in vivo. With the targeted delivery strategy, the antibiotic became active in both settings.

**Figure 4:**
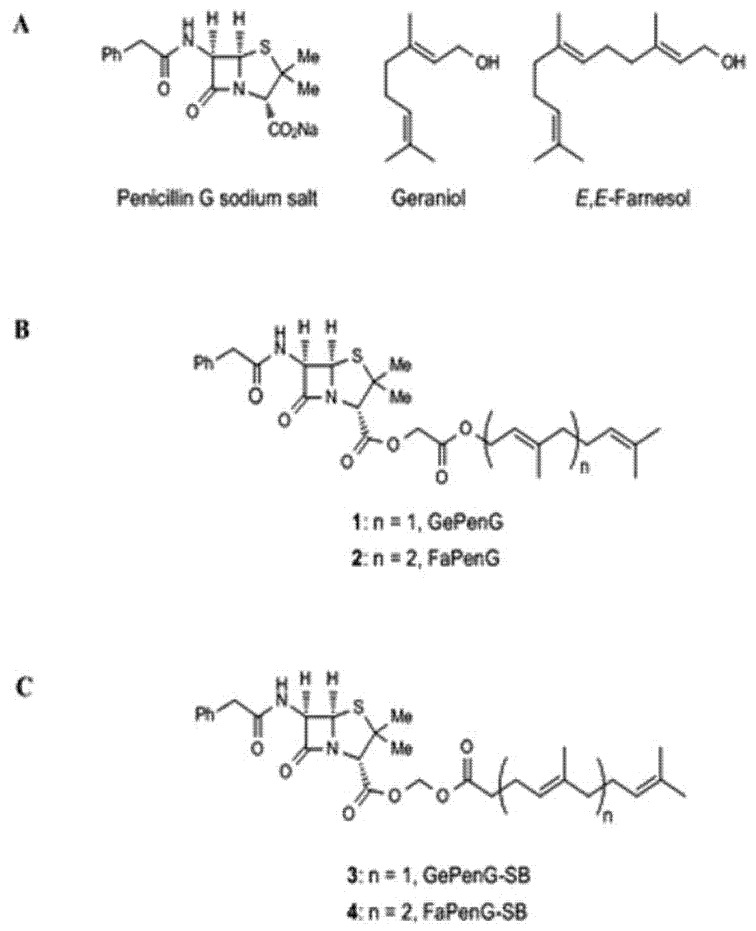
Chemical structures of penicillin G prodrugs using terpenoid moieties for the treatment of *Staphylococcus aureus* (reprinted with permission from Abed et al. [109], licensed under CC BY 4.0, no modifications were made).

Interestingly, one group reported a strategy that could be considered a hybrid of prodrug and nanomedicine approaches. Farnesyl and geranyl moieties were covalently bonded to penicillin G to improve its intracellular antimicrobial efficacy against *Staphylococcus aureus* to address the issue of antibiotic resistance (Figure 4) [109]. Previously, squalene was successfully attached to penicillin G, after which the construct proceeded naturally to self-assembled into nanoparticles [110]. The nanosized material displayed antibacterial efficacy superior to unmodified penicillin G. Unfortunately, it was also considerably toxic to human cells, thereby limiting the utility of the prodrug. It was then hypothesized that farnesol and geraniol, fellow members of the terpenes family as squalene, would pose less of an toxicity issue because of their shorter chain length [109]. Targeting moieties were connected to the drug via an acylal linker. Regardless of the pH level (pH 4.5 and 7.4 tested), the linker was selectively cleaved in the presence of bacterial cell lysate, but remained largely intact in the presence of buffer alone. Using several microscopic techniques, the researchers demonstrated that both kinds of nanoparticles extensively diffused into macrophages within 24 h. However, farnesyl prodrug proved to be more cytotoxic than geranyl counterpart, and thus was not a viable option. Furthermore, the latter managed to eradicate most of the intracellular pathogens within as little as six hours.

Albayati et al. devised a prodrug whereby a bone-targeting moiety was attached to vancomycin to facilitate its accumulation in the bone for more successful treatment of osteomyelitis due to *Staphylococcus aureus* [111]. Absolute resistance to vancomycin by the pathogen is not the underlying reason for clinical failures in this particular case. Rather, highly hydrophilic physicochemical nature of the drug molecule renders it ineffective for diffusion into the bone tissue [112]. As a result, vacomycin concentration local to the bone could be subtherapeutic. The bone-targeting moiety employed was a 3-*O*-phosphate ester conjugate of 17-β-*O*-{1-[2-carboxy-(2-hydroxy-4-methoxy-3-carboxamido)anilido]ethyl}1,3,5(10)-estratriene. It was previously shown to display an excellent binding affinity to hydroxyapatite, which was superior to that of tetracycline [113]. A reconstructed PEG linker was used to bridge the two components. The prodrug experienced a five-fold increase in bone accumulation [111]. At the same time, clearance of the antibiotic significantly decreased, and half-life significantly prolonged. Given the time-dependent behavior of vancomycin activity [114], the ability of the prodrug to remain above the minimum inhibitory concentration (MIC) in the bone for more than 100 hours is undoubtedly a vital asset in ensuring the success of oseteomyelitis treatment [111]. One thing to note is that the authors did not specify whether the bone-targeting moiety will eventually be cleaved from the construct to release vancomycin. Thus, it is possible that this construct might not behave as a prodrug in the strictest sense, and that vancomycin could still eliminate *Staphylococcus aureus* bacteria while still being anchored to the targeting agent.

## 4. Biological Carrier Strategies

### 4.1. Blood Cell

A handful of different cell types have been conceptualized as potential vehicles for transporting drugs, and slowly liberating the therapeutic cargo exclusively at the intended sites [115]. Apart from the common benefits of targeted delivery systems, these carriers of human origin naturally exhibit an excellent level of biocompatibility and biodegradability, and pose minimal immunogenicity issue [116]. On the other hand, this approach suffers from intrinsic inconsistency in drug-loading capacity as compared to non-biological constructs [117]. The carriers, given their biological origin, consume additional efforts and resources for proper storage without compromising their functional integrity [118]. Moreover, this strategy is not universally applicable to every single antibiotic. Encapsulation of clotrimazole rendered the erythrocyte carriers vulnerable to oxidative injuries, thereby making the cells more prone to phagocytosis by hepatic reticuloendothelial system (RES) [119].

Several methods have been proposed for loading the drug into the cell-based carriers such as endocytosis [120], osmotic pulse triggered by dimethyl sulfoxide [121], electroporation [122], anchoring to cell-penetrating peptides [123], and osmotic-based methods (e.g., hypotonic pre-swelling [124] and hypotonic dialysis [125]). An up-to-date account of the methods and the procedure could be found in a recent review [126].

Among the cell types hitherto tested, erythrocyte is the most widely studied [115]. Techniques for cellular drug encapsulation have been around for more than two decades. At an earlier stage, researchers in the field experimented with rat erythrocytes before moving on to human erythrocytes. Talwar and Jain tested numerous methods to entrap metronidazole in rat erythrocytes, attaining a loading efficiency of 42–56% [127]. However, these cellular carriers displayed osmotic vulnerability, and failed to prevent bleeding of their content over time. Within 12 h after encapsulation, 60% and 34% of metronidazole and hemoglobin respectively were present outside the cells. Treatment of the cells with glutaraldehyde partially helped address this leakage issue by fortifying their stability. Glutaraldehyde-treated erythrocytes were shown in a prior in vivo study to not cause any observable harm to the liver even with prolonged exposure [128]. 

The concentration of glutaraldehyde needs to be optimized depending on the intended target site. It was observed that low glutaraldehyde concentrations caused the carriers to be primarily picked up by the spleen RES [129]. Conversely, higher concentrations promoted uptake of the erythrocytes by RES in the liver. Eichler et al. attempted to further increase the RES-targeting capacity of these cellular carriers by anchoring anti-Rh antibodies to the surface of gentamicin-containing erythrocytes [130]. It was suggested that a small cell count per administration coupled with a high antibody density per cell were optimal. To keep the number of erythrocyte carriers low while not compromising the concentration of antibiotic delivered, it is definitely important to ensure an adequate loading capacity. With a similar purpose in mind, Jain et al. tested a different auxiliary strategy by co-encapsulating magnetite alongside isoniazid [131]. An external magnetic field was used to facilitate the localization of the construct. 

As a carrier for targeted delivery, erythrocytes have the advantageous ability to transport their cargo to the RES, become phagocytosed by the macrophages, then release the therapeutic load in a slow-release fashion with zero-order kinetics [132]. Consequently, a greater amount of the antibiotic could reach facultative intracellular pathogens such as the mycobacteria [133]. Using hypotonic dialysis, Millán et al. encased amikacin in human erythrocytes. Loaded erythrocytes performed better in osmotic fragility test than unmodified red blood cells. The authors suggested that the osmotic-based method of encapsulation employed had winnowed out the more fragile group of blood cells. A similar observation had been previously reported [125].

Rossi et al. also applied hypotonic dialysis technique for erythrocytes loading [134]. What separates this study from the ones discussed thus far is that the antimicrobial agent encapsulated was not a traditional antibiotic, but a recombinant hemolytic toxin (i.e., listeriolysin O). The biological carrier facilitated the co-localization of the toxin with the *Mycobacterium avium* complex inside the phagosomes, which proved to be elusive to standard drugs. As a result, the anti-mycobacterial activity of listeriolysin O was greatly enhanced. Yet it failed to completely shut down replication of the pathogen. The authors reasoned that repeated dosing of the toxin-loaded erythrocytes would improve the therapeutic outcome. 

More recently, Baistrocchi et al. employed leukocytes, instead of the popular erythrocytes, as cellular carriers of posaconazole for the treatment of invasive pulmonary aspergillosis [135]. *Aspergillus fumigatus* filamentous hyphae spread and injure pulmonary tissues, resulting in formation of a necrotic perimeter that hinders the penetration of antifungals towards the center of the infection site [136,137]. The specific kind of leukocyte selected was neutrophil. Aside from performing defense mechanism against *Aspergillus fumigatus* [138], neutrophils had previously been demonstrated to efficiently make their way to the pathogen hyphae [139]. These carriers allowed posaconazole to better infiltrate the pathogen hideout where it could showcase its high potency [140]. As such, the fungal burden dropped to a large extent compared to administration of free drug [135].

### 4.2. Bacteriophage

Researchers first appreciated the applicability of bacteriophage in the fight against microbes in the early 20th century [141]. Yet, the dawn of the antibiotics golden era cast too large a shadow for it to really take off. Since then, efforts have been few and far between, leaving the field overgrown with subpar experimental designs and conflicting conclusions regarding the efficacy of the phages [142,143]. Only once we began to acknowledge the encroaching threat known as multi-drug resistance did scientists finally turn their attention back to the once forgotten therapeutic option. One major advantage of bacteriophage over small-molecule antibiotics is its intrinsic specificity to a group of bacterial strains [144]. This attribute allows the phage to avoid inflicting collateral damage on non-targets, be it human cells or commensal bacteria. This apparent forte, however, could also prove to be its shortcoming. Being overly strain-specific, a single kind of phage might not be able to target all the different strains present at the infection site [145]. 

Investigation into the use of bacteriophage as a vehicle for targeted delivery only emerged recently. Vaks and Benhar tethered chloramphenicol prodrug to f1 filamentous coliphage via a hydrophilic linker [146]. More specifically, the native drug molecule was first subjected to chemical reactions to introduce a moiety that would be cleaved by esterases. The drug-binding linker was then conjugated to carboxyl groups present on phage coat proteins. This conjugation step was demonstrated to render the phage more biocompatible and more suitable as a carrier. Conjugated phage barely possessed any infectivity, thereby eliminating a source of concern that this biological carrier might replicate inside host body and cause complications. Although filamentous phage is known for its excessive immunogenicity [147], the authors showed that the conjugation process limited immune recognition by anti-PVIII antibody [146]. Mice injected with chloramphenicol-conjugated phages did not produce antibodies against the prodrug. Moreover, being conjugated to f1 coliphage somehow negated the severe systemic toxicity of the antibiotic, hence rendering it more much tolerable as a viable therapeutic option [148]. In vivo improvement of the antimicrobial efficacy of the native drug was verified against mice infected with *Staphylococcus aureus* [146].

Another group conjugated the chloramphenicol prodrug to filamentous phage [149], but introduced additional targeting capacity to the construct. This new feature took the form of either targeting peptides expressed on major coat proteins, or immunoglobulin G (IgG) expressed on minor coat proteins.

Relative to various other strategies that do not employ nanoparticles for site-specific delivery of antibiotic, conjugation bacteriophage approach is only in its infancy stage. The evidence thus far has suggested that it is an approach with much potential, and definitely deserves more attention from researchers. 

## 5. Future Perspectives

Moving forward, nanomedicine will likely remain the mainstay in the field of targeted delivery of antibiotics, at least in the near future. However, these nanosized materials are not without drawbacks. Although the toxicology of antibiotic-coupled nanoparticles has scarcely been studied, intrinsic adverse health effects of nanoparticles alone are well established [150]. For instance, carbon nanotubes have been shown to trigger apoptosis, necrosis, and oxidative stress in mammalian cells [151,152]. Studies in rats have revealed toxicity of nanoparticles in various organs such as the lung [153], kidney [154], and liver.

Given the downsides of nanomedicine, it is in our best interest to concurrently explore alternative stratagems such as macromolecular conjugates and biological carriers. Being of biological origin, these vehicles have naturally displayed remarkable biocompatibility and biodegradability [115]. Moreover, conjugation of antibiotics to macromolecules has helped improve pharmacokinetic properties of the therapeutic agents [58].

Despite the apparent advancement in the field, there are still challenges to overcome before these systems could be translated from benchtop to bedside. For instance, variation in physicochemical parameters (e.g., amount of antimicrobial molecules per conjugate) is an issue largely associated with the formation of macromolecular conjugates. Meanwhile, biological carriers encounter this problem even further upstream at the cell sources. Alongside a refinement in synthetic processes, it is crucial to devise reliable methods of characterization, both in vitro and in vivo, to help minimize intra- and inter-batch variability, especially with respect to drug loading. After all, a system in which we are incapable of precisely controlling the dose is of limited utility. It also remains to be determined whether manufacturing processes after up-scaling have any deleterious ramification on the bioactivity or stability or biological carriers such as erythrocytes. Even after all these technical issues are tackled, the cost barrier persists. At the current stage, additional costs involved in the manufacturing of either drug conjugates or cellularly carried drugs will undoubtedly render the commercial products less affordable to the lower-income populations.

Having said that, it is important for us to acknowledge that these alternative strategies possess promise, as outlined throughout this article. At a time when the industry pipeline for novel un-resisted antibiotics is limited, targeted delivery systems of antimicrobial agents present a viable solution. Macromolecular conjugate and biological delivery carriers would undoubtedly expand the limited armament in the fight against the escalating threats of multidrug-resistant infections. 

Thus far, there have not been many targeted delivery systems of antibiotics that have made it to clinical trials. Nonetheless, that should not be misconstrued as a lack of applicability of these strategies in clinical practice. Rather, it stems from the relatively underwhelming amount of attention given in the medical community to these non-nanomedicine approaches. TD-1792, a conjugate between glycopeptide and cephalosporin, is currently undergoing clinical trials for the treatment of skin infections and has yielded promising results [155]. Although this antibiotic–glycopeptide conjugate adopts a multivalent ligand mechanism of action and is not exemplary of conjugates devised for targeted delivery, it is undoubtedly a testament to the potential bedside utility of macromolecular conjugates.

Neither macromolecular conjugate nor biological carrier are going to be applied commercially as delivery systems for antimicrobial agents any time soon. Nevertheless, given the right amount of research funding and effort, these strategies could potentially haul us out this pit known as “antibiotic resistance”.
